# Human influenza A H5N1 in Indonesia: health care service-associated delays in treatment initiation

**DOI:** 10.1186/1471-2458-13-571

**Published:** 2013-06-11

**Authors:** Wiku Adisasmito, Dewi Nur Aisyah, Tjandra Yoga Aditama, Rita Kusriastuti, Trihono, Agus Suwandono, Ondri Dwi Sampurno, Prasenohadi, Nurshanty A Sapada, MJN Mamahit, Anna Swenson, Nancy A Dreyer, Richard Coker

**Affiliations:** 1 Universitas Indonesia, Depok, Indonesia; 2 CDC-EH MoH, Jakarta, Indonesia; 3 NIHRD MoH, Jakarta 10560, Indonesia; 4 Persahabatan Hospital, Persahabatan Raya St. No. 1, East Jakarta, Pisangan Timur, Pulo Gadung 13230, Indonesia; 5 Sulianti Saroso Hospital, North Jakarta, Sunter, Indonesia; 6 Tangerang Hospital, Tangerang, Indonesia; 7 Outcome Quintiles, Cambridge, MA, USA; 8 CDPRG, London School of Hygiene and Tropical Medicine, Bangkok, ThailandCDPRG, London School of Hygiene and Tropical Medicine, Bangkok, Thailand

**Keywords:** Influenza A virus H5N1, Delivery of health care, Virulence, Indonesia

## Abstract

**Background:**

Indonesia has had more recorded human cases of influenza A H5N1 than any other country, with one of the world’s highest case fatality rates. Understanding barriers to treatment may help ensure life-saving influenza-specific treatment is provided early enough to meaningfully improve clinical outcomes.

**Methods:**

Data for this observational study of humans infected with influenza A H5N1 were obtained primarily from Ministry of Health, Provincial and District Health Office clinical records. Data included time from symptom onset to presentation for medical care, source of medical care provided, influenza virology, time to initiation of influenza-specific treatment with antiviral drugs, and survival.

**Results:**

Data on 124 human cases of virologically confirmed avian influenza were collected between September 2005 and December 2010, representing 73% of all reported Indonesia cases. The median time from health service presentation to antiviral drug initiation was 7.0 days. Time to viral testing was highly correlated with starting antiviral treatment (p < 0.0001). We found substantial variability in the time to viral testing (p = 0.04) by type of medical care provider. Antivirals were started promptly after diagnosis (median 0 days).

**Conclusions:**

Delays in the delivery of appropriate care to human cases of avian influenza H5N1 in Indonesia appear related to delays in diagnosis rather than presentation to health care settings. Either cases are not suspected of being H5N1 cases until nearly one week after presenting for medical care, or viral testing and/or antiviral treatment is not available where patients are presenting for care. Health system delays have increased since 2007.

## Background

Human Avian Influenza represents a major public health concern due to the high case fatality rate of the disease coupled with the potential for novel influenza viruses to cause pandemics. By 21^st^ February 2012, 586 cumulative cases have been reported globally. Of these, Indonesia has reported more than any other country. The cumulative number of cases reported from Indonesia by the same date is 185, or 32% of the world’s cases. Globally the case fatality rate associated with influenza A H5N1 infection is high, with 346 deaths (59%) reported from confirmed cases. In Indonesia the case fatality rate is 83% (153/185) [1]. We recently reported the impact of treatment on the clinical course of influenza H5N1 and showed that though treatment with oseltamivir within 48 hours offers significant benefits in terms of survival; the benefits of treatment persist in terms of reduced case fatality rates, though to a lesser extent, even if treatment is delayed up to 6 to 8 days after symptom onset [2,3]. Given the very high mortality rates associated with H5N1 infection in Indonesia and the known association with delays in initiating treatment, it is important to determine whether delays occur across the country or whether some provinces experience longer delays than others, and whether delays are the result of patients delaying seeking health care or whether delays in the health care service itself are important.

The purpose of this study is to determine whether delays in treatment in Indonesia result from delays in presentation for care, subsequent virological testing, or the timing of the initiation of antiviral drug treatment by health care providers. We also investigate the association between province location, demographic and epidemiological characteristics, symptoms at presentation, and delays in care. To the best of our knowledge this is the first analysis of health service barriers to care for human H5N1 cases.

## Methods

Data on human avian influenza (H5N1) cases were collected between September 2005 and December 2010. Most case information came from the Indonesia Ministry of Health, Provincial and District Health Office clinical records. The characteristics of some cases were obtained from literature, and added to registry [4].

Cases were identified through routine surveillance reports, supplemented by reviews of hospital and clinic records and laboratory reports. Cases were defined accordingly to the World Health Organization case (WHO) guidelines, and through laboratory confirmation from both Indonesia and WHO laboratories. Data were collected retrospectively and entered into a reporting registry [5]. This database included patient characteristics, clinical, laboratory, exposure, treatment and outcome information.

For each case the following timelines were determined: date of symptom onset, date of first presentation to medical care, date samples were taken for viral testing, and date of initiation of antiviral drug treatment. The site of first presentation for medical care was characterized as one of the following: emergency room, physician’s office/clinic, rural health service/public health centre, or other setting. Patient characteristics include age, sex, clinical characteristics of initial disease manifestation, and history of likely source of exposure to possible sources of infection with H5N1. Since data were collected retrospectively from existing data sources, the degree of completeness was dependent upon the presence of data in the source record. All signs and symptoms documented were analysed.

The registry protocol was approved by the ethical review committee of the University of Indonesia and also by a central institutional review board in the United States.

### Statistical analysis

Cases with missing data on either the time intervals of interest or the exposure (including cases where the exposure was coded as “not known or not documented”) were excluded from analyses. The denominator for each analysis is the number of cases with non-missing values for the relevant variable. Since time intervals were not normally distributed, nonparametric statistical tests were used. For comparisons between two groups the Wilcoxon Rank Sum test was used; for three groups or more the Kruskal-Wallis test was used. If the p-value for a Kruskal-Wallis test was ≤ 0.05, then pair-wise comparisons between groups were conducted to identify which pairs were significantly different. A Bonferroni correction for multiple comparisons was used when reporting results of pair-wise comparisons. The Spearman rank-order correlation test was used to examine the relationship between two continuous variables. Chi-square test was used to test the association between categorical variables.

## Results

This report is based on 124 laboratory confirmed human avian influenza H5N1 cases for which data were collected between 1^st^ September 2005 – 31^st^ December 2010. Table 1 summarizes demographic characteristics, presenting clinical characteristics, and history of exposure to possible sources of infection with H5N1. These cases represent 73% of all Indonesia cases reported to WHO over the same time period. We were unable to retrieve data through case record retrieval on the remaining 27% of cases.

**Table 1:** Demographic and epidemiological characteristics

**Demographic and epidemiological characteristics**	**Analysis population (n = 124)**
**Sex***(% male)*	54 (43.6%)
**Age***(median, range)*	19.5 (1.5-67)
**Exposure***n(%)*	
Any human exposure	8 (6.5%)
Direct poultry exposure	43 (34.7%)
Indirect poultry exposure	29 (23.4%)
Direct wild bird exposure	1 (0.01%)
Indirect wild bird exposure	5 (4.0%)
In vicinity of live poultry	55 (44.4%)
**Time from symptom onset to presentation for medical care (Days)***– median (range)***n = 114**	0 (0-7)
**Time from presentation for medical care to viral testing (Days) *–****median (range) ***n = 94**	6 (-4.0 -25.0)
**Time from presentation for medical care to antiviral treatment(Days) *–****median (range)***n = 43**	7 (1.0-11.0)

Location of first presentation for medical care was unknown for 6 cases. Of the 118 cases with known location, 55% first presented for medical care to a physician’s office and 21% to an emergency room; the remainder presented elsewhere. Case fatality appears to differ by type of health care facility where cases first presented (p = 0.06) (Table 2). Those presenting to rural health service and public health centers had the lowest case fatality rate.

**Table 2:** Case fatality rates by location of presentation for medical care

**Location of first presentation for medical care**	**N (%)**	**Case fatality rate (p = 0.06)**
Emergency room	25 (21%)	22/25 (88%)
Physician’s office or clinic	65 (55%)	61/65 (94%)
Rural health service/public health center	17 (14%)	12/17 (71%)
Other*	11 (9%)	10/11 (91%)
All**	118 (100%)	105/118 (89%)

*“Other” includes nurse (n = 4) and midwife (n = 7).

**6 cases have unknown location of presentation and are not included in this table Two of the six cases died; the other four survived.

### Delays in viral testing and initiation of treatment

Data on time from symptom onset to presentation at medical care provider were available for 114 cases, of which 94 cases presented to medical care and underwent viral testing, and 43 cases who received antiviral treatment. The median time from symptom onset to presentation was zero days with a range of zero to 7 days. Although there were no significant differences for the time from symptom onset to presentation for medical care between the various types of health care providers (Table 3), time to viral testing was significantly different by provider type (p = 0.04). Overall cases waited 6 days from presentation for medical care to viral testing. Pair-wise testing showed that the only statistically significant difference in time to viral testing was between the emergency room and “Other” groups (p = 0.01), with patients presenting to other healthcare providers (consisting of nurses and midwives) experiencing an average wait of 3.5 days longer than those presenting at an emergency room. The median time from viral testing to antiviral treatment was zero days suggesting that viral testing was coincident with treatment initiation across all health care providers. Time from presentation for medical care to viral testing (n = 94), and presentation for medical care to antiviral treatment (n = 43), were highly correlated (Spearman’s correlation = 0.88, p < 0.0001). However, none of the pair-wise comparisons of case fatality rate by health care facility were statistically significantly different.

**Table 3:** Timing of events by location of first presentation for medical care

	**Symptom onset to presentation for medical care**		**Presentation for medical care to viral testing**		**Presentation for medical care to antiviral treatment**	
**Location of presentation for medical care**	N (%)	Median days (Min, Max)	N (%)	Median days (Min, Max)	N (%)	Median days (Min, Max)
**Emergency room**	24(21)	0.0 (0.0, 7.0)	20 (21)	5.0 (-4.0, 10.0)*	6 (14)	6.5 (2.0, 8.0)
**Physician’s office or clinic**	62(54)	0.0 (0.0, 7.0)	51 (54)	6.0 (0.0, 25.0)	25 (58)	7.0 (1.0, 10.0)
**Rural health service/public health center**	17(15)	0.0 (0.0, 7.0)	15 (16)	6.0 (1.0, 11.0)	8 (19)	5.0 (2.0, 11.0)
Other**	11(10)	0.0 (0.0, 4.0)	8 (9)	8.5 (3.0, 9.0)	4(9)	8.0 (5.0, 10.0)
**Total**	114	0.0 (0.0, 7.0)	94	6.0 (-4.0, 25.0)	43	7.0 (1.0, 11.0)
		*p* = 0.82		***p*** **= 0.04**		*p* = 0.68

*Significantly different from other. One case reportedly had a sample drawn for viral testing 4 days before their first reported presentation for medical care. Since this case was part of a cluster of human cases, it is plausible that the case was tested during an epidemiological investigation and before presenting for treatment, however, this could not be confirmed. **“Other” includes nurse (n = 4) and midwife (n = 7).

No significant difference in time from presentation for medical care to treatment with oseltamivir (an influenza-specific antiviral drug) was seen by health care provider type for the 43 cases for whom data were available. The median duration from presentation to oseltamivir initiation was 7.0 days (minimum 1.0 days, maximum 11.0 days, Table 3).

### Province location and delays

We analyzed delays in presentation, viral testing and treatment initiation and compared these across eight Indonesian provinces from which cases were reported. We found no statistically significant associations with delays according to province.

### Demographic and clinical associations with delays

Neither age nor sex was significantly associated with any delays in presentation, access to viral testing, or treatment initiation. With regard to influenza H5N1 from infected humans, wild birds, and/or poultry, a reduced time to treatment initiation approached statistical significance (p = 0.06) only for cases who were exposed to other human cases of H5N1 disease. Of 30 documented signs and symptoms on presentation, two were associated with delays in both presentation and viral testing: unexplained respiratory illness and tachypnoea (Table 4). No signs or symptoms were associated with significantly reduced delays in treatment initiation.

**Table 4:** Timing of events by symptom at presentation for medical care

	**Symptom onset to presentation for medical care**			**Presentation for medical care to viral testing**			**Presentation for medical care to antiviral treatment**		
**Symptom noted at first presentation for medical care**	N	Median days (Min, Max)	*p*-value	N	Median days (Min, Max)	*p*-value	N	Median days (Min, Max)	*p*-value
**Fever**	93	0.0 (0.0, 7.0)	0.34	76	6.0 (-4.0, 18.0)	0.21	38	6.5 (1.0, 11.0)	0.11
**No fever**	8	0.0 (0.0, 2.0)		8	7.5 (1.0, 25.0)		2	9.5 (9.0, 10.0)	
**Unexplained respiratory illness**	66	0.0 (0.0, 7.0)	**0.05**	57	6.0 (0.0, 25.0)	**0.04**	20	7.0 (1.0, 11.0)	0.71
**No unexplained respiratory illness**	38	0.0 (0.0, 4.0)		32	7.0 (3.0, 18.0)		22	7.0 (2.0, 11.0)	
**Excessive sputum**	1	0.0 (0.0, 0.0)	0.61	1	7.0 (7.0, 7.0)	0.97	1	7.0 (7.0, 7.0)	0.80
**No excessive sputum**	50	0.0 (0.0, 7.0)		45	7.0 (0.0, 25.0)		26	8.0 (1.0, 11.0)	
**Sore throat/pharyngitis**	25	0.0 (0.0, 7.0)	0.06	19	5.0 (-4.0, 11.0)	0.14	12	6.0 (1.0, 11.0)	0.69
**No sore throat/pharyngitis**	44	0.0 (0.0, 7.0)		42	7.0 (0.0, 25.0)		22	7.0 (2.0, 10.0)	
**Rhinorrhea**	2	0.5 (0.0, 1.0)	0.73	2	6.5 (6.0, 7.0)	0.77	2	7.5 (7.0, 8.0)	0.83
**No rhinorrhea**	66	0.0 (0.0, 7.0)		57	6.0 (0.0, 25.0)		33	7.0 (1.0, 11.0)	
**Tachypnea**	27	1.0 (0.0, 7.0)	**0.04**	23	5.0 (-4.0, 10.0)	**< 0.01**	6	6.0 (1.0, 11.0)	0.40
**No tachypnea**	64	0.0 (0.0, 7.0)		57	7.0 (1.0, 25.0)		31	7.0 (2.0, 11.0)	
**Cyanosis**	0	-	N/A	0	-	N/A	0	-	N/A
**No cyanosis**	56	0.0 (0.0, 7.0)		49	6.0 (0.0, 11.0)		32	7.0 (2.0, 11.0)	
**Abnormal breath sounds**	0	-	N/A	0	-	N/A	0	-	N/A
**No abnormal breath sounds**	40	0.0 (0.0, 5.0)		36	7.0 (0.0, 11.0)		25	8.0 (2.0, 11.0)	
**Diarrhea**	9	0.0 (0.0, 2.0)	0.43	7	6.0 (3.0, 8.0)	0.70	1	9.0 (9.0, 9.0)	0.47
**No diarrhea**	70	0.0 (0.0, 7.0)		60	6.0 (0.0, 18.0)		33	7.0 (1.0, 11.0)	
**Abdominal pain**	14	0.5 (0.0, 7.0)	0.20	11	6.0 (3.0, 9.0)	0.89	5	7.0 (5.0, 9.0)	0.98
**No abdominal pain**	60	0.0 (0.0, 7.0)		53	6.0 (0.0, 18.0)		32	7.0 (1.0, 11.0)	
**Vomiting**	11	0.0 (0.0, 1.0)	0.91	8	6.5 (3.0, 10.0)	0.43	4	8.0 (7.0, 9.0)	0.43
**No vomiting**	70	0.0 (0.0, 7.0)		62	6.0 (0.0, 25.0)		32	7.0 (1.0, 11.0)	
**Headache**	25	1.0 (0.0, 7.0)	**< 0.01**	21	6.0 (0.0, 9.0)	0.65	8	7.0 (5.0, 10.0)	0.84
**No headache**	48	0.0 (0.0, 7.0)		41	7.0 (0.0, 25.0)		26	7.5 (1.0, 11.0)	
**Neurological involvement**	0	-	N/A	0	-	N/A	0	-	N/A
**No neurological involvement**	53	0.0 (0.0, 7.0)		46	7.0 (0.0, 25.0)		28	7.5 (2.0, 11.0)	
**Psychiatric symptoms**	0	-	N/A	0	-	N/A	0	-	N/A
**No psychiatric symptoms**	43	0.0 (0.0, 5.0)		38	7.0 (0.0, 11.0)		27	8.0 (2.0, 11.0)	
**Fatigue**	11	1.0 (0.0, 4.0)	0.21	10	6.0 (3.0, 7.0)	0.32	4	6.0 (3.0, 7.0)	0.18
**No fatigue**	58	0.0 (0.0, 7.0)		49	7.0 (0.0, 18.0)		29	7.0 (1.0, 11.0)	
**Myalgia**	6	0.5 (0.0, 2.0)	0.38	5	7.0 (6.0, 9.0)	0.47	2	6.0 (5.0, 7.0)	0.49
**No myalgia**	51	0.0 (0.0, 7.0)		43	7.0 (0.0, 11.0)		27	8.0 (1.0, 11.0)	
**Bleeding gums or nose**	0	-	N/A	0	-	N/A	0	-	N/A
**No bleeding gums or nose**	80	0.0 (0.0, 7.0)		69	6.0 (-4.0, 25.0)		35	7.0 (1.0, 11.0)	
**Enlarged Liver**	0	-	N/A	0	-	N/A	0	-	N/A
**No enlarged liver**	45	0.0 (0.0, 4.0)		37	7.0 (1.0, 11.0)		27	8.0 (2.0, 11.0)	
**Conjunctivitis**	0	-	N/A	0	-	N/A	0	-	N/A
**No conjunctivitis**	59	0.0 (0.0, 7.0)		51	7.0 (0.0, 25.0)		30	7.0 (2.0, 11.0)	

### Temporal changes in delays in presentation, viral testing, and initiation of treatment

Cases who presented with influenza H5N1 in 2005 to 2007 (n = 73) took slightly longer to present for medical care than cases who developed disease later (n = 51). Conversely, the time from presentation to a health care facility to viral testing and also for treatment initiation was shorter for cases who were infected in 2005 to 2007 compared with those infected in more recent years (Table 5).

**Table 5:** Timing of events by year of infection

	**Symptom onset to presentation for medical care**		**Symptom onset to viral testing**		**Symptom onset to antiviral treatment**	
**Year of Infection**	N (%)	Median days (Min, Max)	N (%)	Median days (Min, Max)	N (%)	Median days (Min, Max)
**2005-2007 (n = 73)**	63(86)	1.0 (0.0, 7.0)	47(64)	5.0 (-4.0, 25.0)	14	5.0 (1.0, 11.0)
**2008-2010 (n = 51)**	51(100)	0.0 (0.0, 7.0)	47(92)	7.0 (1.0, 11.0)	29	7.0 (2.0, 11.0)
		*p* = 0.007		*p* = 0.022		*p* = 0.012

## Discussion

This study represents the first analysis of health system delays in the clinical management of human H5N1 influenza and shows that, in Indonesia, delays result largely because of health service limitations rather than patients delaying seeking care. We found that delays in treatment are principally a function of health services rather than delays in patients seeking care. Even in rural settings, patients present soon after symptoms develop which suggests that public awareness campaigns regarding H5N1 and the need for early presentation hve been successful. Early treatment with oseltamivir is recommended, and delays beyond 48 hours after symptom onset result in worse outcomes [3,6,7]. We found that the median time from health service presentation to antiviral drug initiation was 7.0 days in Indonesia, and case fatality rates remain high despite the use of oseltamivir treatment. The delayed initiation of treatment appears to be an important contributor to the especially high case fatality rates documented from Indonesia [8].

Delays in the initiation of influenza specific antiviral drugs may be the result of either a lack of drugs at sites of care or a lack of clinical suspicion of H5N1 by the treating health care workers. The latter seems most likely, and concurs with other reports suggesting better clinical outcomes in patients who are part of clusters of cases where clinical suspicion is likely to be high [4]. Furthermore, H5N1 case-finding has not focused on the identification of asymptomatic or mild cases of H5N1 virus infection [4,9-11]. Moreover, the Indonesian Ministry of Health has distributed stockpiles of drugs to all provincial health offices, referral hospitals, and other hospitals. Seven million capsules of oseltamivir have already been distributed to health center level in accordance with the Indonesia Centre for Disease Control (CDC) guidelines [12].

A low clinical suspicion of disease by health care workers likely remains an important impediment to early diagnosis, virological confirmation, and appropriate treatment initiation [13]. The signs and symptoms during the first two days of disease in cases reported here were mostly non-specific. This nonspecific clinical presentation of influenza A (H5N1) disease raises challenges. The differential diagnosis of cases may include other influenza-like illnesses, dengue, or typhoid [14], to the exclusion of influenza A (H5N1). In an earlier report, only 12% of influenza H5N1 cases were initially diagnosed as having influenza H5N1 [13].

Early treatment is important in achieving clinical success and a high index of clinical suspicion is necessary for patients presenting, sometimes with non-specific symptoms, to clinical settings. For any benefits to accrue from prompt presentation for medical care, clinical suspicion needs to be raised amongst physicians and other care-givers. Whilst patients presented earlier for medical care prior to 2008, perhaps because of the significant investments in community awareness of avian influenza [15], increasing delays in viral testing and the initiation of appropriate treatment, irrespective of type of health care setting, appear to be increasing. The late health care seeking behaviour in 2005-2007 may have resulted from a low level of awareness in population [16]. By 2009, research suggests, community awareness in Indonesia had increased [17].

We found that those presenting to rural health services and public health centres had lower fatality rates than those presenting to other sites of care (Table 2). This was a surprising finding. A possible explanation may be that patients with an obviously poor prognosis may have been referred to higher level health facilities without being formally admitted to rural health centres or public health centres.

There are some limitations in our study. Whilst we analysed data from 124 out of 171 cases, we could not collect data for the remaining cases due to administrative challenges. Since the missing cases mostly are from the island of Sumatra, these results may not be generalizable to health care services delays in a a different geographic area.

Although we found that the median time from presentation to antiviral drug initiation was 7.0 days, some questions remain unanswered. For example, we have insufficient data to determine which health care providers are limited in their capacity to provide prompt treatment for patients, and why. Most patients (55%) first seek medical treatment at a physician’s office or clinic and go to the hospital when the disease becomes severe. But we are not able to offer insights into how the movement of patients between health care settings influences care provision in terms of delay in initiation of treatment. Nonetheless, we believe our analyses provide important insights on delays in presentation from symptom onset, to viral testing, and to initiation of treatment in Indonesia.

## Conclusions

Reducing health care system delays in the initiation of specific treatment for patients infected with influenza H5N1 is no easy matter. The non-specific nature of the disease, especially in the early days, suggests a number of options that might be considered. The application of rapid diagnostic tests on presentation to confirm or refute the diagnosis might enable clinicians to tailor their treatment better. Alternatively, the initiation of treatment when clinical suspicion is raised might offer benefits to the minority who actually have influenza H5N1. Both of these approaches have cost implications that need to be determined. Prospective clinical studies too may offer more robust data on clinical symptoms and signs associated with differentiating H5N1 from other diseases as well as determining those likely to fare least well clinically and thus benefit most from influenza specific clinical interventions.

## Competing interests

Professor Adisasmito received modest support to facilitate data collection and review. Professor Coker has received grant funding from F. Hoffmann-La Roche, the manufacturer of oseltamivir. Dr. Dreyer and Ms. Swenson are employed by Quintiles Outcome, a company, that specializes in patient registries and which has received funding from Hoffmann-La Roche to design and maintain a global registry of human H5N1 cases.

## Authors’ contributions

WA reviewed this manuscript’s intellectual content, provided important insights of the research, and gave final approval for the version to be published. DNA assisted data collection and contributed to drafting the manuscript. TYA provided expert information on Indonesian AI cases and reviewed manuscript. RK provided clinical and epidemiological data information and reviewed manuscript. TH provided NIHRD data information and reviewed manuscript. AS linked network information between NIHRD and CDC-EH Ministry of Health and reviewed manuscript. ODS shared data information of AI cases and reviewed manuscript. PH, NAS, and MJNM provided AI cases data information from hospitals and reviewed manuscript. AS contributed to the execution of the study and writing the paper. ND led the study design, execution and analysis, and contributed to writing the manuscript. RC conceived the research question, conceptualized the research design, and contributed to drafting the paper. All authors read and approved the final manuscript.

## Pre-publication history

The pre-publication history for this paper can be accessed here:

http://www.biomedcentral.com/1471-2458/13/571/prepub
